# Supplementary material for: Stepwise heating in Stille polycondensation toward no batch-to-batch variations in polymer solar cell performance
Source: Nat Commun. 2018 May 14;9:1867. doi: 10.1038/s41467-018-03718-7 (PMC5951883; doi:10.1038/s41467-018-03718-7)
Supplement: Supplementary file 1 — Supplementary Information(PDF 786 kb) [file 41467_2018_3718_MOESM1_ESM.pdf]

Supplementary Information

Lee *et al*. Stepwise heating in Stille polycondensation toward no batch-to-batch variations in polymer solar cell performance

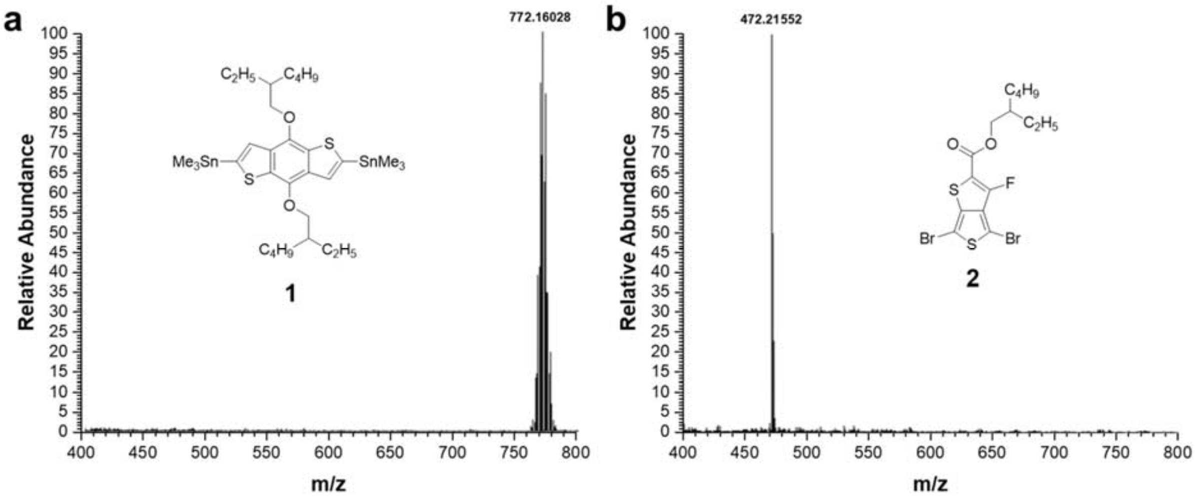

Supplementary Figure 1. High resolution mass spectra of monomer **1** and **2** for (**a)** and (**b**), respectively

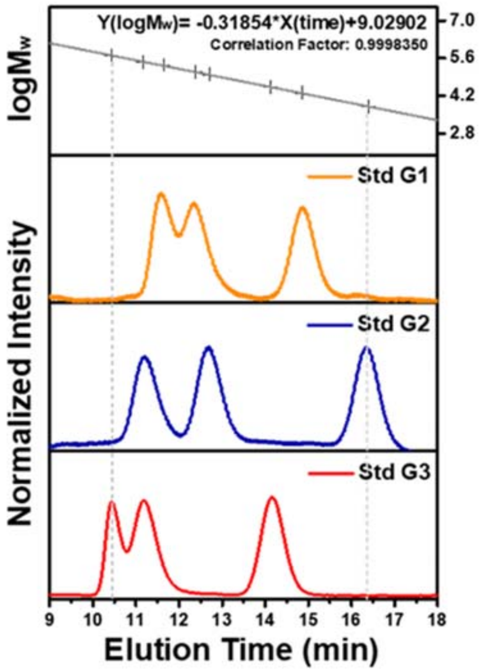

Supplementary Figure 2. GPC profiles of the broad ranges of the polystyrene standards and the calculated calibration points and line with the equation and the correlation factor.

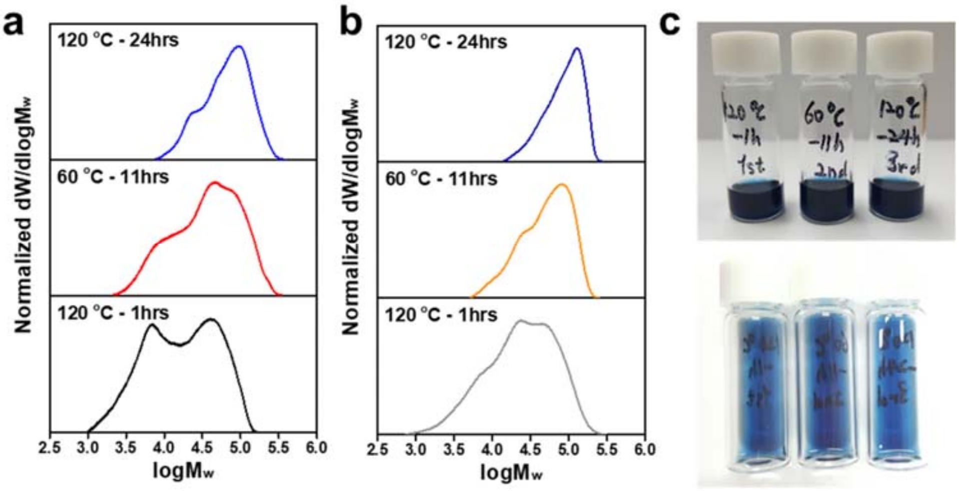

Supplementary Figure 3. GPC profiles of time-dependent growing trends of polymeric chains by $\log M_w$ in conventional Stille polycondensation for entries 1 and 10 (**a**) and in the stepwise method for entry 11 (**b**) with pictures of a set of three steps clearly dissolved in toluene/DMF for entry S9 (**c**).

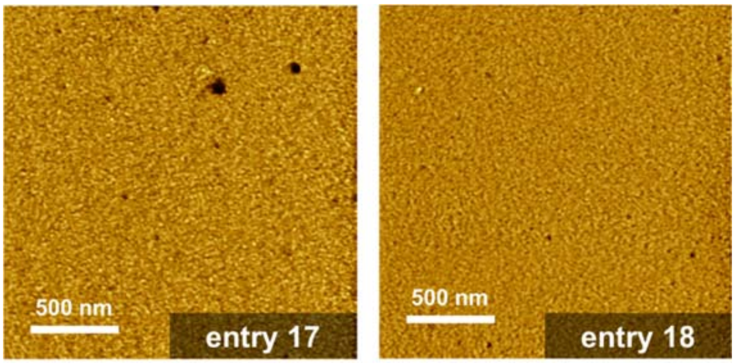

Supplementary Figure 4. AFM phase images of entries 17 and 18.

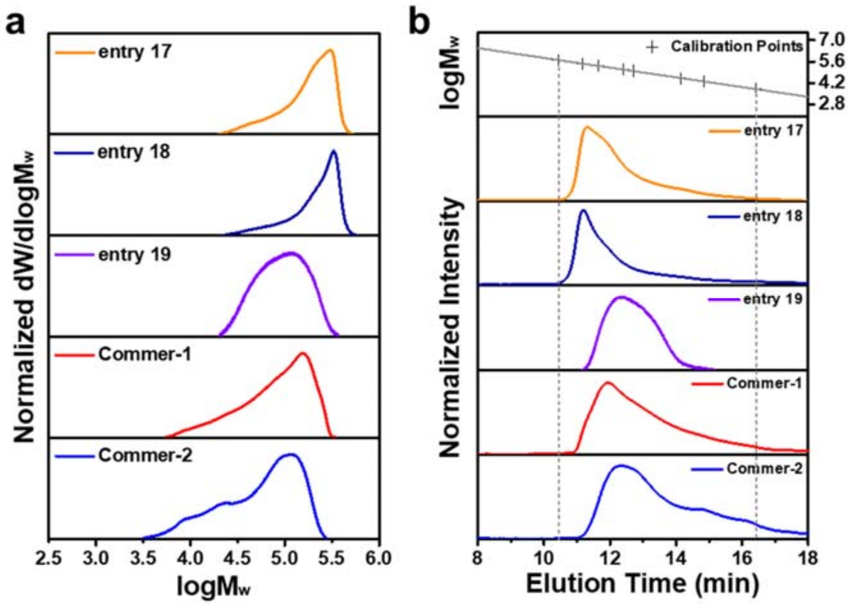

Supplementary Figure 5. GPC plots of Figure 3 in weight distribution mode (**a**) and with the calibration points and lines (**b**).

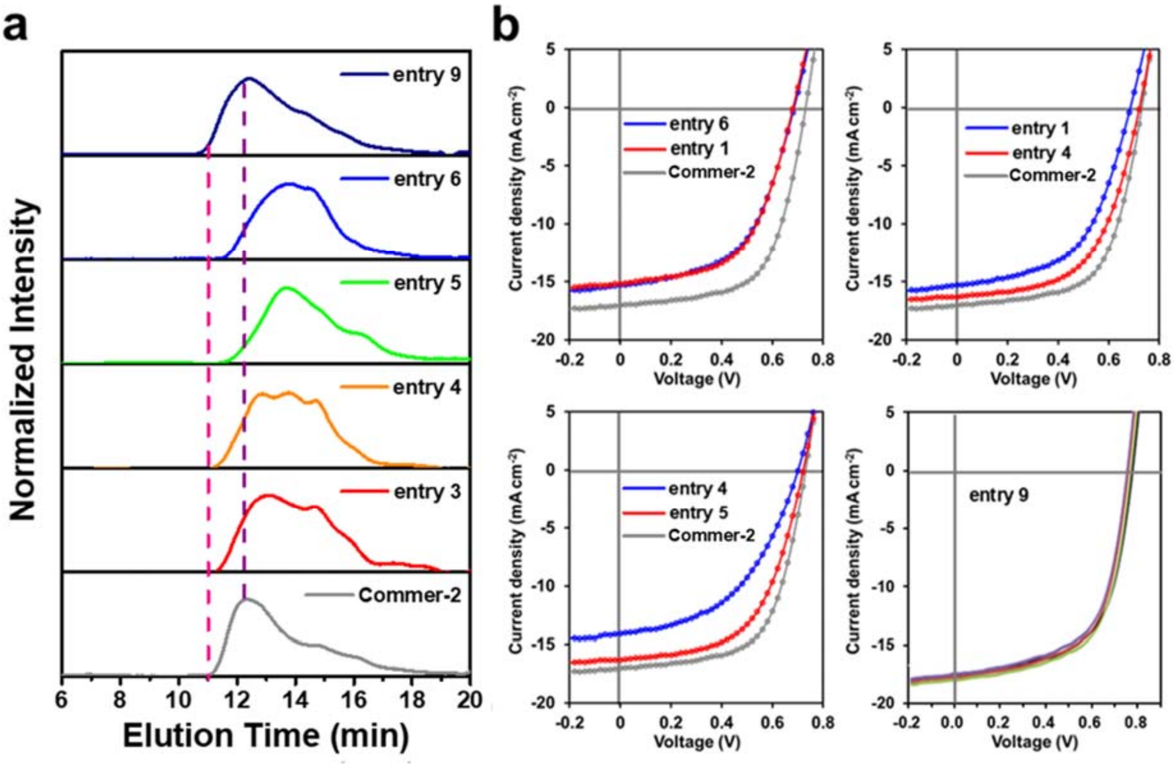

Supplementary Figure 6. GPC profiles of entries 3, 4, 5, 6, 9, and Commer-2 (**a**), and *J–V* characteristics of solar cell devices fabricated using the selected entries (**b**).

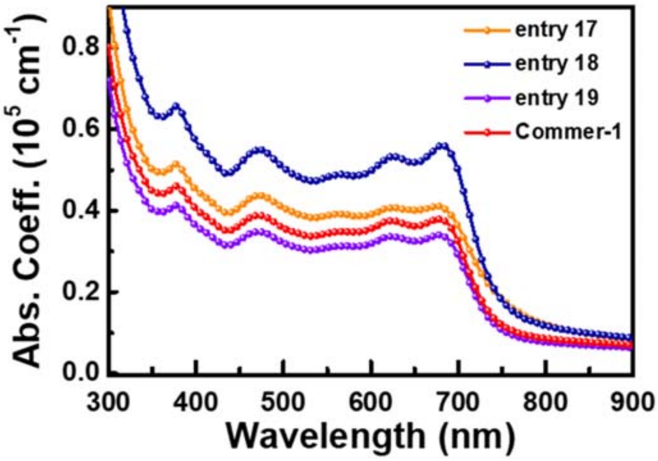

Supplementary Figure 7. UV-vis spectra of the blending films with $PC_{71}BM$ (1:1.7 *wt*%).

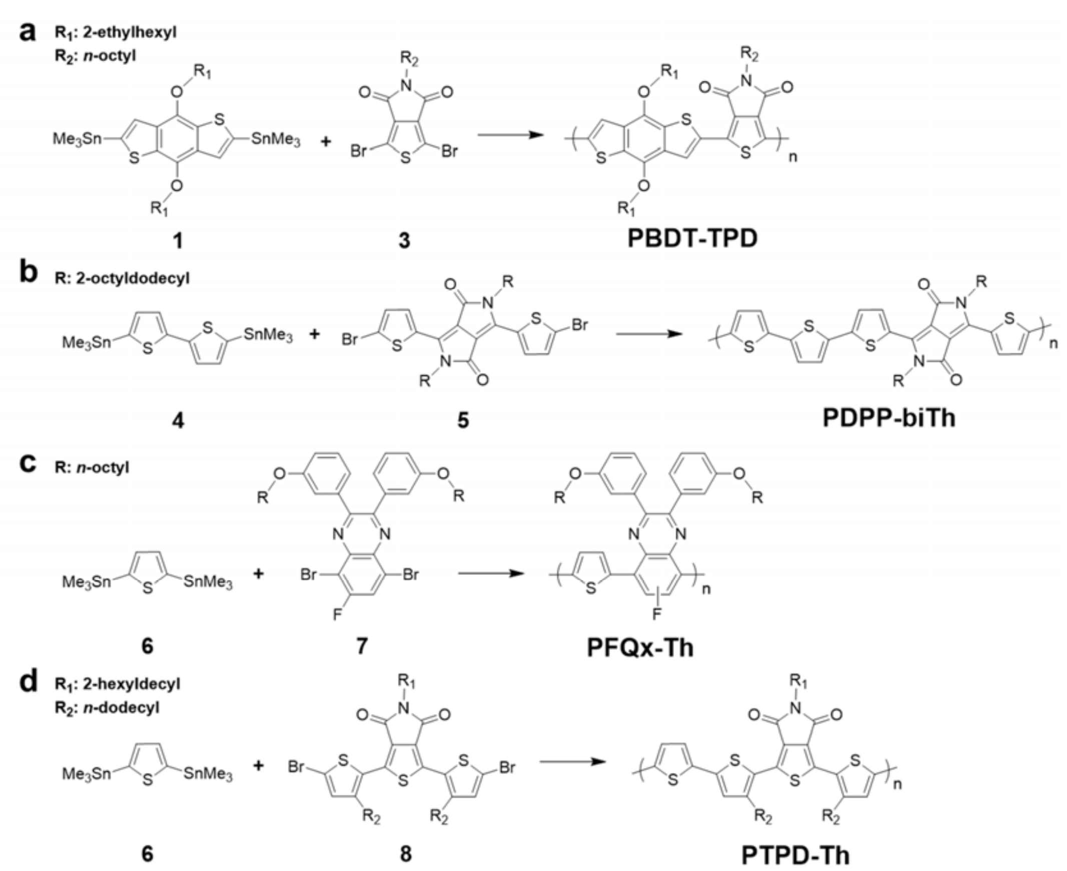

Supplementary Figure 8. Synthetic routes for PBDT-TPD (**a**), PDPP-biTh (**b**), PFQx-Th (**c**), and PTPD-Th (**d**).

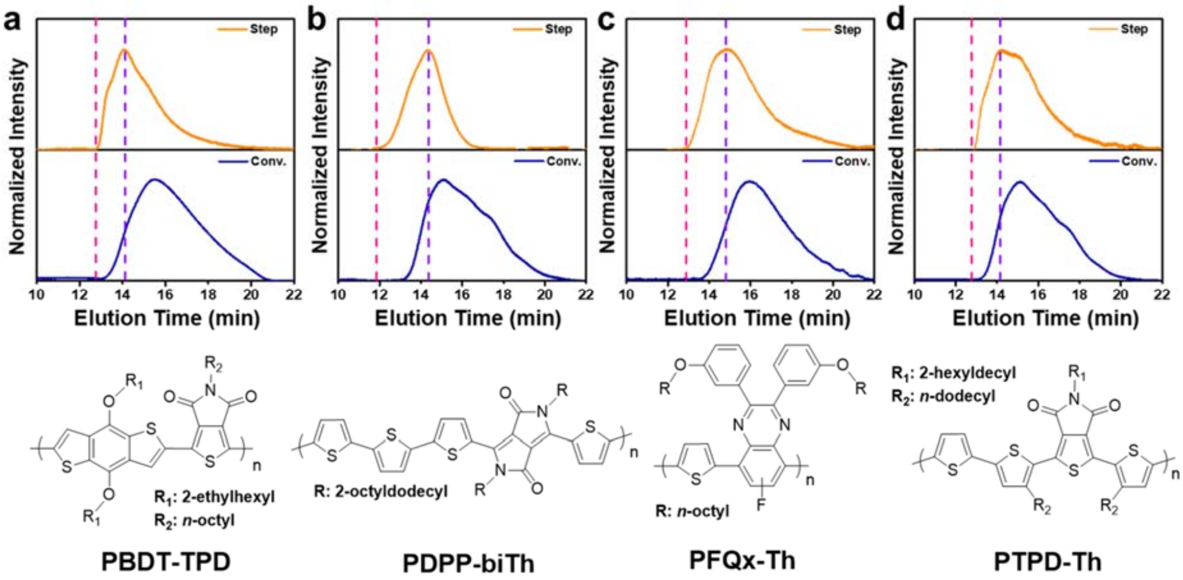

Supplementary Figure 9. Comparison of stepwise and conventional Stille polycondensations by GPC profiles and structures for PBDT-TPD (**a**), PDPP-biTh (**b**), PFQx-Th (**c**), and PTPD-Th (**d**).

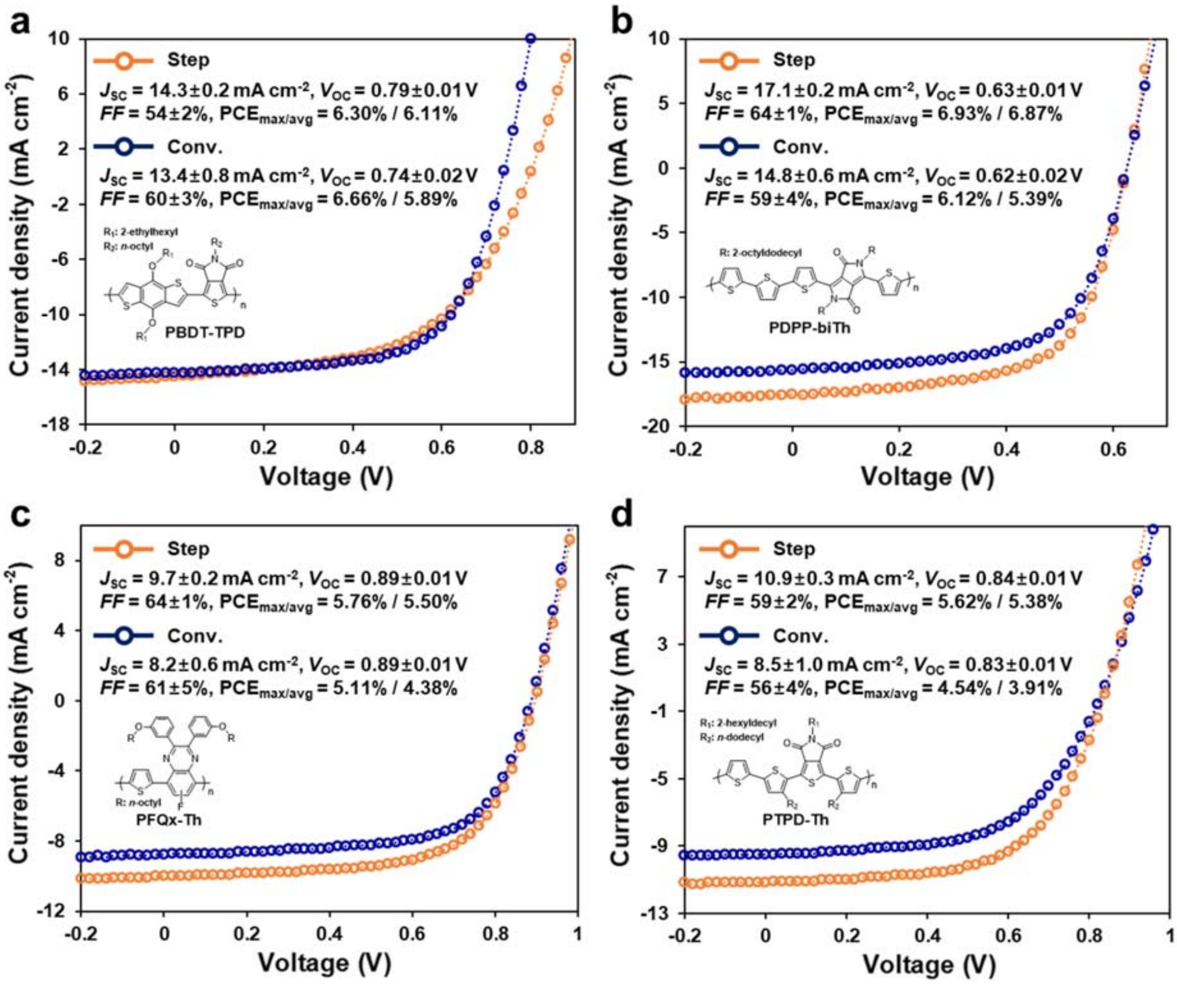

Supplementary Figure 10. Comparison of *J*–*V* characteristics with stepwise and conventional batches for PBDT-TPD (**a**), PDPP-biTh (**b**), PFQx-Th (**c**), and PTPD-Th (**d**).

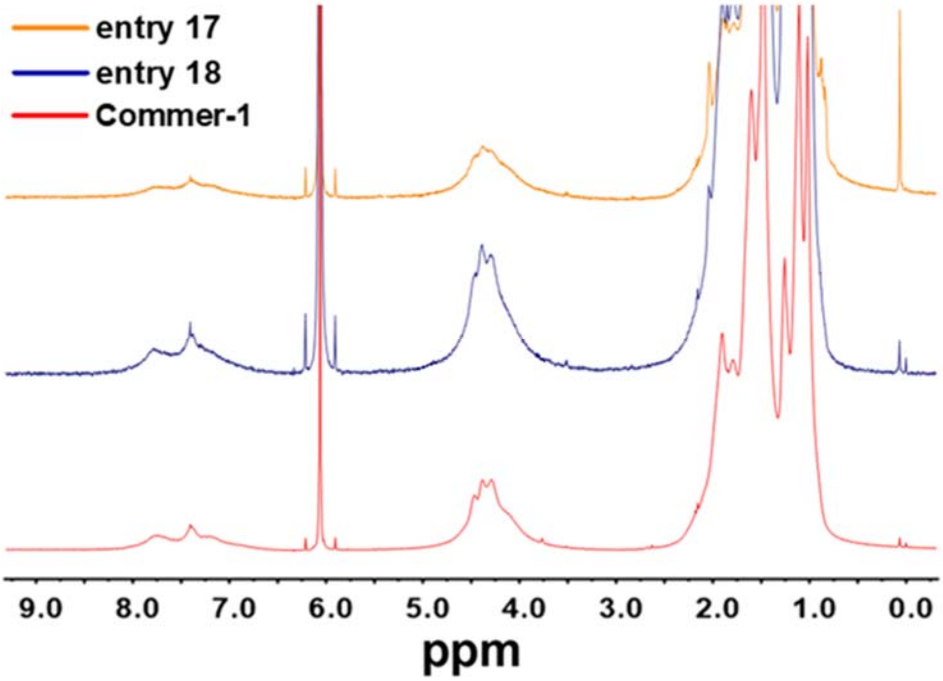

Supplementary Figure 11. High-temperature ^{1}H NMR spectra of entries 17, 18 and Commer-1.

Supplementary Table 1. Elemental analyses and mass information of monomer **1** and **2**.

Monomer	Calculated				Found			
	**carbon**	**hydrogen**	**sulfur**	**m/z (ESI)**	**carbon**	**hydrogen**	**sulfur**	**m/z (ESI)**
1	49.76	7.05	8.30	772.16032	49.82	7.13	8.30	772.16028
2	38.15	3.63	13.58	471.90003	38.21	3.65	13.64	472.21552

.

.

Supplementary Table 2. The standard groups of monodisperse polystyrenes used for GPC calibrations with their polymeric parameters.

Standards	M_w (kDa)	logM_w	Elution Time$_{max}$ (min)
Standard Group 1 (Std G1)	217	5.34	11.65
	127	5.09	12.39
	20.1	4.30	14.85
Standard Group 2 (Std G2)	282	5.45	11.17
	96.1	4.98	12.71
	6.31	3.80	16.41
Standard Group 2 (Std G3)	508	5.71	10.43
	282	5.45	11.17
	32.9	4.52	14.13

Supplementary Table 3. The additional experiments for the screenings in the conventional Stille polycondensation.

**Entry**	**Changes in the method**	M_n **(kDa)**[*]	M_w **(kDa)**[*]	**Đ**[*]
S1	injection method[†]	4.50	7.10	1.60
S2	-	23.3	41.3	1.76
	post-polymerization[‡]	26.1	47.3	1.94
S3	-	16.6	33.7	2.03
	post-polymerization[§]	19.9	41.0	2.06

The conventional Stille polycondensation was carried out under an argon atmosphere in a long Schlenk tube of monomer **1** and **2** in 0.10 M solution, and 4 mol% of $Pd(PPh_3)_4$. [*]M_n, M_w, and *Đ* values were determined from GPC measurement using 1,2,4-trichlorobenzene at 120 °C calibrated with polystyrene as standard. [†]An auto-injector was employed for dropwise of the solution of the ditin monomer in toluene. [‡]Post-Stille polycondensation of the extracted chloroform fraction noted in above row was carried out with an excess of compound **1** in same conditions for 12 h. [§]Direct arylation polymerization of the extracted fraction in hexane was carried out with an excess of compound **2** in the condition of an arbitral amount of $Pd(OAc)_2$ and pivalic acid in THF with K_2CO_3 at 80 °C for 12 h.

Supplementary Table 4. The additional GPC results of each set of batches in stepwise Stille polymerization varying the temperatures and the catalyst loadings.

Entry	Temp. (°C)	Time (h)	M_n (kDa)[*]	M_w (kDa)[*]	$Đ$[*]
S4[†]	60	4	12.6	17.4	1.38
	90	4	21.6	31.3	1.45
	120	24	42.4	75.0	1.77
11[‡]	120	1	14.8	40.6	2.75
	60	11	36.7	62.1	1.69
12[‡]	120	1	11.0	28.7	2.61
	80	11	34.0	71.7	2.11
13[‡]	120	1	9.15	27.2	2.97
	100	11	45.1	88.4	1.96
S5[§]	Stepwise Polymerization		70.1	101	1.44
S6[‖]	120	1	26.4	55.2	2.09
	60	11	27.9	56.1	2.01
	120	24	103	137	1.33
S7[‖]	120	1	19.2	38.7	2.02
	60	11	30.1	57.9	1.93
	120	24	79.7	126	1.58
S8[‖]	120	1	42.6	81.4	1.91
	60	11	82.8	120	1.45
	120	24	143	188	1.31
S9[‖]	120	1	46.4	84.5	1.82
	60	11	87.9	124	1.41
	120	24	151	190	1.25

The stepwise Stille polycondensation was carried out under an argon atmosphere in a long Schlenk tube of monomer **1**, **2** and $Pd(PPh_3)_4$ and procedures included the initial heating at 120 °C for 1 h, the cooling step at 60 °C for 11 h, and the final heating at 120 °C for 1 day. [*]M_n, M_w, and $Đ$ values were determined from GPC measurement using 1,2,4-trichlorobenzene at 120°C calibrated with polystyrene as standard. [†]The gradual increasing in temperature was attempted including at 60 °C for 4 h, 90 °C for 4 h, and finally 120 °C for 1 day. [‡]The cooling temperature was investigated as followed by Table 2 in manuscript at 60 °C, 80 °C, and 100 °C for entries 11, 12, and 13, respectively. [§]Different catalyst system, $Pd_2(dba)_3$/P(*o*-tolyl)$_3$ (2/8 mol%) was adopted. [‖]The catalyst loadings were initially varied from 4.0 mol% to 1.0 mol%.

Supplementary Table 5. The optical and electrical properties of entries 17 and 18.

Polymers	λ_{max}^{film} (nm)*	ε_{abs}^{film} (cm^{-1})†	E_g^{opt} (eV)‡	E_{HOMO} (eV)§	E_{LUMO} (eV)§	E_g^{elec} (eV)ǀ
entry 17	627, 679	93,900	1.68	-5.34	-3.61	1.73
entry 18	627, 679	133,000	1.67	-5.34	-3.61	1.73

*Polymer films were spin-coated on glass substrates. †The absorption coefficient (ε) was obtained using the equation ($\varepsilon = 2.303 \times (A/l)$), where A is the absorbance and l is the film thickness (in cm). ‡The optical band gap was determined from the onset of the UV-vis absorption spectra in the polymer films. §Energy levels are measured by cyclic voltammetry with Fc/Fc^+ (E_{HOMO} = -4.80 eV) as the internal reference. ǀThe electronic band gap was calculated from $E_g^{elec} = E_{LUMO} - E_{HOMO}$.

Supplementary Table 6. Crystallographic parameters calculated from GIWAXD profiles of thin-films of entries 17 and 18.

**Crystallographic Parameters**		**entry 17**	**entry 18**
q (Å^{-1})	q_{xy} (100)	0.321	0.317
d-spacing (Å)		19.8	19.6
FWHM (Å^{-1})		0.149	0.150
Correlation length (Å)		37.8	37.6
q (Å^{-1})	q_z (010)	1.58	1.60
d-spacing (Å)		3.95	3.93
FWHM (Å^{-1})		0.336	0.298
Correlation length (Å)		17.1	19.3

Supplementary Table 7. The polymeric parameters of commercially purchased samples.

Company	M_n (kDa)*	M_w (kDa)*	*Đ**
Commer-1	56.2	109	1.94
Commer-2	36.6	82.4	2.25

*M_n, M_w, and *Đ* values were determined from GPC measurement using 1,2,4-trichlorobenzene at 120 °C calibrated with polystyrene as standard.

Supplementary Table 8. The photovoltaic parameters for the conventional Stille batches with Commer-2.

**Samples**	J_{SC} **(mA cm^{-2})**	V_{OC} **(V)**	***FF*** **(%)**	**PCE (%)**
entry 1	14.45	0.72	56	5.77
entry 4	16.31	0.72	56	6.65
entry 5	14.09	0.70	48	4.73
entry 6	13.79	0.69	57	5.40
entry 9	17.89	0.73	59	7.74
Commer-2	17.02	0.73	62	7.73

Supplementary Table 9. The results of GPC measurements for additional four polymers synthesized by stepwise and conventional methods.

Polymers	Temp. (°C)	Time (h)	M_n (kDa)*	M_w (kDa)*	$Đ$*	Yield (%)†
PBDT-TPD	120	1	8.21	18.1	2.19	-‡
	60	11	23.2	43.1	1.87	-‡
	120	24	91.3	127	1.40	73
	Conventional Polymerizations[1]		30.1	64.2	2.14	82
PDPP-biTh	100	-§	11.4	34.4	3.02	-‡
	60	11	27.4	59.6	2.17	-‡
	100	24	81.2	101	1.26	88
	Conventional Polymerizations[2]		37.7	71.8	1.91	85
PFQx-Th	140	2	12.5	29.9	2.40	-‡
	60	10	33.9	71.4	2.11	-‡
	140	48	82.1	112	1.36	54
	Conventional Polymerizations[3]		27.3	51.5	1.88	69
PTPD-Th	120	1	8.28	11.8	1.43	-‡
	60	11	21.2	34.0	1.60	-‡
	120	24	98.6	139	1.41	89
	Conventional Polymerizations[4]		40.5	74.8	1.85	57

The conventional batches were carried out following the cited papers. *M_n, M_w, and $Đ$ values were determined from GPC measurement using 1,2,4-trichlorobenzene at 120 °C calibrated with polystyrene as standard. †Yields were estimated from the amounts of the chloroform fractions. ‡Each fraction was extracted by a syringe, then precipitated in methanol with Soxhlet purification for only GPC analysis. §In the optimized conditions for PDPP-biTh case, the initial step was proceeded until the solution color was changed to green.

Supplementary Note 1: Optimized Stepwise polycondensation for PBDT-TPD, PDPP-biTh, PFQx-Th, and PTPD-Th.

Stepwise Stille polycondensation of poly[4,8-bis[(2-ethylhexyl)oxy]benzo[1,2-b:4,5-b']dithiophene-2,6-diyl-alt-5-octylthieno[3,4-c]pyrrole-4,6-dione-1,3-diyl] (PBDT-TPD): To a mixture of monomers **1** (100 mg, 0.13 mmol) and **3** (54.8 mg, 0.13 mmol) in a binary mixture of toluene and DMF (0.10 M) as a 4:1 ratio, the solution of $Pd(PPh_3)_4$ in toluene (2.6 mM) was injected with subsequent argon purging for 30 min. The long Schlenk tube was placed in a preheated oil bath at 120 °C for 1 h for the initial stage, after which allowed the temperature to reduce and kept the tube cooled for 11 h. The reaction temperature was set again to 120 °C and continuously maintained for 1 day. Cooling down to room temperature, the precipitates in methanol was purified and the fraction from chloroform was characterized under the same process conditions used for the PTB7.

Stepwise Stille polycondensation of poly[2,5-bis(2-octyldodecyl)pyrrolo[3,4-c]pyrrole-1,4(2H,5H)-dione-3,6-diyl-alt-2,2':5',2'':5'',2'''-quaterthiophene-5,5'''-diyl] (PDPP-biTh): To a mixture of monomers **4** (48.3 mg, 0.098 mmol) and **5** (100 mg, 0.098 mmol) in toluene (0.078 M), the solution of $Pd(PPh_3)_4$ in toluene (2.0 mM) was injected with subsequent argon purging for 30 min. The long Schlenk tube was placed in a preheated oil bath at 100 °C during color change to green for the initial stage, after which allowed the temperature to reduce and kept the tube cooled for 11 h. The reaction temperature was set again to 100 °C and continuously maintained for 1 day. Cooling down to room temperature, the precipitates in methanol was purified and the fraction from chloroform was characterized under the same process conditions used for the PTB7.

Stepwise Stille polycondensation of poly[6-fuoro-2,3-bis-(3-octyloxyphenyl)quinoxaline-5,8-diyl-alt-thiophene-2,5-diyl] (PFQx-Th): To a mixture of monomers **6** (57.3 mg, 0.14 mmol) and **7** (100 mg, 0.14 mmol) in chlorobenzene (0.11 M), $Pd_2(dba)_3/P(o\text{-tolyl})_3$ (1.4/5.6 μmol) was added with subsequent argon purging for 30 min. The long Schlenk tube was placed in a preheated oil bath at 140 °C for 2 h for the initial stage, after which allowed the temperature to reduce and kept the tube cooled for 10 h. The reaction temperature was set again to 140 °C and continuously maintained for 2 days. Cooling down to room temperature, the precipitates in methanol was purified and the fraction from chloroform was characterized under the same process conditions used for the PTB7.

Stepwise Stille polycondensation of poly[5-(2-hexyldodecyl)thieno[3,4-c]pyrrole-4,6-dione-1,3-diyl-alt-4',4''-didodecyl-2,2':5',2''-terthiophene-5,5''-diyl] (PTPD-Th): To a mixture of monomers **6** (39.5 mg, 0.097 mmol) and **8** (100 mg, 0.097 mmol) in a binary mixture of toluene and DMF (0.039 M) as a 4:1 ratio, $Pd_2(dba)_3/P(o\text{-tolyl})_3$ (1.4/5.6 μmol) was added with subsequent argon purging for 30 min. The long Schlenk tube was placed in a preheated oil bath at 120 °C for 1 h for the initial stage, after which allowed the temperature to reduce and kept the tube cooled for 11 h. The reaction temperature was set again to 120 °C and continuously maintained for 1 day. Cooling down to room temperature, the precipitates in methanol was purified and the fraction from chloroform was characterized under the same process conditions used for the PTB7.

Supplementary Note 2: Device Fabrications of PBDT-TPD, PDPP-biTh, PFQx-Th, and PTPD-Th.

The devices based on PBDT-TPD, PDPP-biTh, PFQx-Th, and PTPD-T polymers were fabricated under the similar process conditions used for the PTB7-based devices, except for the followings: chlorobenzene/1,8-diiodooctane solvent (97:3 *vol*%, 8 mg mL^{-1} concentration), polymer:$PC_{71}BM$ (1:1.5 *wt*%) blend ratio, and spin-coating at 1500 rpm for PBDT-TPD; dichlorobenzene/chloroform solvent (1:4 *vol*%, 8 mg mL^{-1} concentration), polymer:$PC_{71}BM$ (1:1.5 *wt*%) blend ratio, and spin-coating at 3000 rpm for PDPP-biTh; dichlorobenzene/chloroform solvent (4:1 *vol*%, 8 mg mL^{-1} concentration), polymer:$PC_{71}BM$ (1:1.5 *wt*%) blend ratio, and spin-coating at 1000 rpm for PFQx-Th; chloroform/1,8-diiodooctane solvent (98:2 *vol*%, 10 mg mL^{-1} concentration), polymer:$PC_{71}BM$ (1:2 *wt*%) blend ratio, spin-coating at 3000 rpm for 1 min, and annealing at 120 °C for 10 min for PTPD-Th, respectively.

Supplementary References

1. Zhang, Y., *et al*. Efficient polymer solar cells based on the copolymers of benzodithiophene and thienopyrroledione. *Chem. Mater.* **22**, 2696-2698 (2010).

2. Liu, F., *et al* Efficient polymer solar cells based on a low bandgap semi-crystalline DPP polymer-PCBM blends. *Adv. Mater.* **24**, 3947-3951 (2012).

3. Dutta, G. K., *et al*. Synthesis of fluorinated analogues of a practical polymer TQ for improved open-circuit voltages in polymer solar cells. *Polym. Chem.* **5**, 2540-2547 (2014).

4. Guo, X., *et al*. Polymer solar cells with enhanced fill factors. *Nat. Photonics* **7**, 825-833 (2013).
